# EGG DWPack: System for Multi-Channel Electrogastrographic Signals Recording and Analysis

**DOI:** 10.1007/s10916-018-1035-1

**Published:** 2018-09-17

**Authors:** Dariusz Komorowski

**Affiliations:** Faculty of Biomedical Engineering, Department of Biosensors and Processing of Biomedical Signals, Silesian University of Technology, Zabrze, Poland

**Keywords:** Electrogastrography, EGG, Analysis software, Matlab

## Abstract

Electrogastrography (EGG) is a non-invasive examination method for investigating the myolectrical activity of a stomach. Nowadays, abdominal surface electrogastrography is the one of methods of stomach examination that is used for diagnosing patients with chronic intractable nausea, vomiting and gastroparesis. The electrogastrographic signals are recorded by using cutaneous electrodes placed on the stomach surface. EGG DWPack system is a highly developed and easy to use software package for four channel electrogastrography recording and analysis. The part of the software for analysis is a MATLAB based software and requires the specific ASCII format of the EGG data. The analyzed EGG signals could be conditioned with the wide range of sampling frequency and various resolutions of analog to digital conversion. Additionally, if the EGG data fulfills certain conditions associated with sampling frequency, the software can be used to study the basic parameters of heart rate variability (HRV) simultaneously with the EGG parameters. The software includes different digital filters for the EGG signal extraction and tools for artifacts exclusion. The software computes the majority of EGG parameters which are commonly used in a clinical practice. The EGG analysis can be made for several adjustable analysis settings and various methods, and it can optimize the analysis methods for different preferences or requirements. The analysis result can be saved in a MAT-file, and exported to MS Excel and ASCII files. Validation of the software was performed using synthetic and real EGG signals. This paper contains, as an example of use, an analysis of four synthetic, and fourteen human 4-channel EGG data recording with water, yogurt and a solid meal stimulation. The mean values of the dominant frequency for fast, and postprandial stage were found to be 2.96±0.21 cpm (cycle per minute), and 3.05±0.33 cpm, respectively. The values established in the validation process are consistent with typical human physiological values. In addition, the results were compared to outcomes from commercial system. The results of validation have proved that EGG DWPack software produces reliable outcomes. The software is available for free of charge for Windows operating system for the all possible non commercial use.

## Introduction

The stomach is electrified and has its own pacemaker which generates electrical events (slow waves) with a frequency of about 3 cycles per minute (cpm) [14, 15, 31, 44, 48]. Gastric motility is regulated by gastric myoelectrical activity that consists of gastric slow waves and spike or second potentials [8, 14]. The gastric slow waves determine the propagation and maximum frequency of gastric contractions [7]. The cutaneous EGG includes only slow waves and disturbance potentials, and usually does not include spikes. This phenomenon is caused by the human abdominal wall, which behaves like a low-pass filter and attenuates higher harmonics of signals [41, 48]. Nowadays, abdominal surface electrogastrography is the one of methods of stomach examination that is used for diagnosing patients with chronic intractable nausea, vomiting and gastroparesis [6, 7, 12, 22, 26–28, 36, 46, 48]. The typical recording process usually takes about 2 hours and consists of a 30-minute pre-prandial part (before the standardized meal), a meal part (when the examined subject eats a standardized meal), and two or three 30-minute postprandial parts. The standard meal depends on the examining center. The typical range of frequency for the EGG signal is from 0.5 to 9.0 cpm (0.008–0.15 Hz) and its amplitude is about 50–400 *μV* [5, 14, 29]. The EGG signal recording procedure is described in detail by Jieyun Yin et all in [48] and by Levanon et all [23]. The analysis of the EGG signal can not be performed by visually examining the electrogastrogram. This is because the EGG signal is the sum of all gastric slow waves presented in the stomach and in addition the recorded signal consists of not only the pure EGG but can include signals from other organs (e.g. respiration signals, colon signal, electrocardiographic signal (ECG), movement artifacts and other disturbances) [36, 48]. The EGG analysis is based on spectral analysis and is performed by computer applications. The spectral analysis of EGG signals is used to determine the frequency distribution of an EGG data fragment (segment). The typical EGG examination is divided into mentioned above parts (often called periods). Each period is usually divided into segments containing 60 to 240 seconds of data. The power spectrum density (PSD) is calculated for each segment. The overall power spectrum density (OPSD) is calculated as an average PSD of all segments for the whole examination and for each period. Based on the PSD and OPSD the dominant frequencies and dominant powers are calculated for the each segment and for the whole examination, and for the each period (overall dominant frequencies (ODF) and overall dominant powers (ODP)). The dominant frequency is defined as a value of frequency for the highest peak (dominant power) of the PSD in the range of 0.5–9.0 cpm [14, 29, 37, 48]. The clinically established parameters are: fasting-fed power ratio, percentage of normal gastric slow waves, percentage of gastric dysrhythmias, percentage of power distribution, and percentage of slow waves coupling [14, 29, 32, 37]. These parameters are calculated based on the DF, DP, ODF and the ODP values. A detailed description of these parameters is included in the next part of that article and can be found in the literature [14, 29, 37].

In this paper, the author introduces the EGG DWPack software (ver.2.0), which is an easy to use EGG analysis tool including a wide variety of analysis options. The tool is a MATLAB based software package.

## Methods

This section introduces the method of processing the EGG signal and analyzed parameters which are commonly used in clinics and included in EGG DWPack software. The computation and descriptions of the parameters are mainly based on the information given in Polygram NET^TM^ reference manual [29], the article written by Parkman H.P. et all; “Electrogastrography: a Document Prepared by The Gastric Section of The American Motility Society Clinical GI Motility Testing Task Force” [37], and given in literature [5, 14, 48].

### The EGG recording

**Fig. 1:**
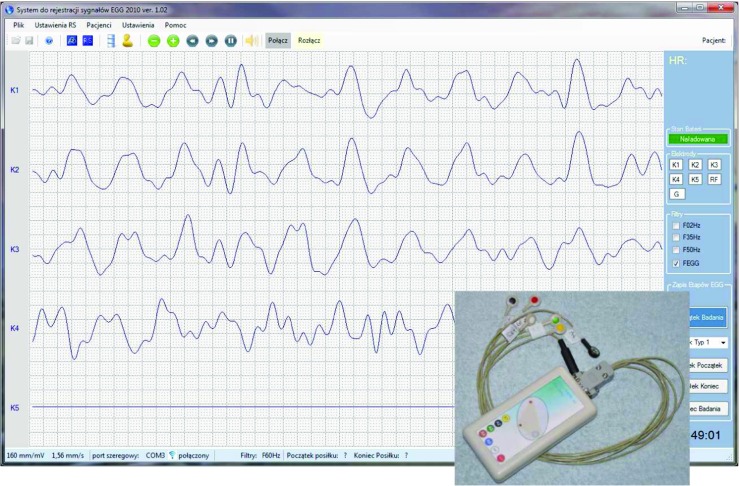
The application for recording the EGG signals and the wireless EGG amplifier (bottom right corner)

The EGG DWPack software package includes the application for recording the EGG signals, but due to the fact that this application requires a specific wireless biomedical amplifier [20] and can not be easily used by the others, this paper only show a view of it and a picture of the wireless EGG amplifier (Fig. 1).

### EGG preprocessing

Due to fact that the EGG signal is rather weak because the amplitude ranges from 50–400 *μV* and the frequency range is between 0.5 to 9.0 cpm (0.008–0.15 Hz) [14], and it is recorded from a stomach surface, it can include a signal from other human organs and a lot of artifacts [9, 23, 24]. The analysis of the EGG signal requires appropriate preprocessing; which includes proper filtering to extract the EGG signal from the recorded signal and attenuate noise components. Also the other artifacts (e.g. caused by patient’s movements) should be removed using the appropriate methods. A proper selection of filtering method and the frequency range is very important to obtain a high quality, undistorted EGG signal. Incorrect preprocessing can cause distortion or even disappearance of gastric slow waves in the EGG [38, 48]. It also can influence computed EGG parameters. The presented software is able to filter the recorded signals by means of three types of band-pass digital filters: the fourth-order zero-phase inverse Butterworth filter [33], fourth-order Butterworth filter and the index blocked Discrete Cosine Transform filter (IB-DCTF) [17]. For each available filter, the frequency range could be changed from 0 to 0.5 Hz (0–30 cpm), and the filtering process could be repeated to choose the proper quality of the EGG signal. In some cases the signal could be recorded with a relatively high sampling frequency (FS) [40], and the resulting signal should be down-sampled to the new lower frequency (e.g. FS= 4 Hz). However, the relatively high sampling frequency is used to simultaneously record the EGG and the non-standard ECG signals (non-standard EGG leads), which are used to analyze the mutual interaction between different human systems (e.g. simultaneously analyze the EGG and HRV examinations) [4, 10, 25, 28, 39, 40]. Before the down-sampling process, the EGG signal must be filtered with a low-pass anti-aliasing filter [3, 33]. The EGG DWPack software can analyze signals recorded with a sampling frequency greater than or equal to 4 Hz. If the FS is above 4 Hz, the signal is filtering by means of anti-aliasing low-pass digital fourth-order Butterworth filter with a cut-off frequency set to 2.0 Hz and down-sampled with the new FS equal to 4 Hz. The filtering process is conducted in both the forward and reverse directions to ensure zero-phase [33]. Two methods are used to find the artifacts and both are based on the analysis of the standard deviation of the amplitude of the EGG signal. These methods are used independently for each channel of the EGG signal.

Method 1; The detection of artifacts is carried out independently in each channel. The signal is divided into segments, for each segment the standard deviation value is determined. Then, the average value of the standard deviation from the segments is calculated. The calculated standard deviation mean value is multiplied by the threshold value (set in the program, default value = 4.2). The calculated value is determined as the artifact threshold. Then, the absolute value of each EGG signal sample is compared to the threshold value of the artifacts. If the absolute value of any signal sample in a given segment exceeds the threshold value, the analyzed segment is marked as excluded from the analysis (bad segment). Exclusion is applied to all channels.
Method 2; The detection of artifacts is carried out independently in each channel. The signal is divided into segments, for each segment the standard deviation value is determined. Then, the average value of the standard deviation from the segments is calculated. The calculated standard deviation mean value is multiplied by the threshold value (set in the program, default value = 1.6). The calculated value is determined as the artifact threshold. Then, the standard deviation value of each segment is compared to the threshold value of the artifacts. If the standard deviation of a given segment exceeds the threshold value, the analyzed segment is marked as excluded from the analysis (bad segment). Exclusion is applied to all channels.

The presented software can set the threshold for finding artifacts and choose a method for removing them. The segments of the EGG signal which include artifacts are marked and are not considered during further analysis.

### Computing the EGG parameters – running spectra analysis

The Running Spectrum Analysis (RSA) provides information about the frequency contents of the myoelectrical signal [29]. The EGG signal is divided into 60 to 256 seconds segments. Except the first segment, each of the segments includes overlap (a few seconds of signal data from the previous segment). The spectrum analysis is made for these segments of the EGG signal. The spectrum analysis of the EGG signal can be performed by a few different methods: the identification parameters of an autoregressive model (AR) and estimation of the PSD, using the periodogram method using the Tukey window [13] (*α* is equal to 0.25), the periodogram method using the Barth-Hanning window, the periodogram method using the Hanning window, or combinations of the AR method with different filters (the available methods are listed in the help). In the AR modeling techniques, the most important parameter is the model order. In the presented software the model order is chosen by using the Akaike information criterion [2]. The selected order of the model substantially influences the PSD shape. If the order is too low, then details of the analyzed signal may be lost; if the order is too high, the power spectrum may contain additional components that do not exist. The model order is calculated for each segment of signal multiplied by the window. To enhance the details of the PSD, the computed model orders are increased by adding a constant value. This value is set to 6 [18]. Next, the PSD is calculated for all segments by means of the chosen method and the values of dominant frequency (DF) and dominant power (DP) are computed. For some shapes of the PSD, the maximum of the PSD occurs at the zero frequency. In this case, the next maximum is analyzed. If the next maximum exists in the range of 0.5 to 9.0 cpm and the difference between the first and the second maximum is less then 2.5 dB, then the DF is established (corrected) for the second maximum, otherwise the segment is classified as arrhythmia [14, 29]. The clinically meaningful parameters of the EGG are calculated based on the values of the DF and DP. These parameters are summarized in Table 1, and detailed definitions and descriptions of them are comprehensively presented in the literature [14, 26, 29, 34, 37, 48].

**Table 1:** The list of EGG parameters (based on RSA) calculated by EGG DWPack software

		Range of application		
Parameters	Units	Segment	Periods	Whole
Dominant frequency	cpm	+	−	−
Mean of dominant frequency	cpm	−	+	+
Median of dominant frequency	cpm	−	+	+
Standard deviation of dominant frequency	cpm	−	+	+
Dominant power	dB	+	−	−
Mean of dominant power	dB	−	+	+
Median of dominant power	dB	−	+	+
Standard deviation of dominant power	dB	−	+	+
Percentage of Bradygastria	a.u. or %	−	+	+
Percentage of normal	a.u. or %	−	+	+
Percentage of Tachygastria	a.u. or %	−	+	+
Percentage of Arrhythmia	a.u. or %	−	+	+
Power instability coefficient	a.u.	−	+	+
Frequency instability coefficient	a.u	−	+	+
Maximum dominant frequency difference	cpm	−	+	+
Percentage of Slow wave coupling	%	−	+	+
Spatial dominant power difference	dB	−	+	+
Percentage distribution of EGG power in the Bradygastria area	%	+	+	+
Percentage distribution of EGG power in the normal area	%	+	+	+
Percentage distribution of EGG power in the Tachygastric area	%	+	+	+

### Computing the EGG parameters – overall spectra analysis

The Overall Spectrum Analysis (OSA) is performed on each single EGG channel. The overall power spectrum density (OPSD) is calculated by averaging the PSDs of one or 4-minute-long segments. For one-minute segments, the overlap was set to 10 seconds, while for four-minute long segments, the overlap is set to 120 seconds. For computing the OPSD, the PSDs are calculated using the autoregressive model (for segments from 1 to 4 minutes in length) and the periodogram (for four minute long segments) methods. The OPSD is calculated for the whole EGG examination and for each of the 30-minute periods. Similarly to calculating the DF and DP, the overall dominant frequency (ODF) and overall dominant power (ODP) are determined [29, 48]. The clinically meaningful parameters of EGG based on OPSD are summarized in Table 2.

**Table 2:** The list of EGG parameters (based on OSA) calculated by EGG DWPack software

		Range of application		
Parameters	Units	Period	Whole	Method of calculating OPSD
Overall DF	cpm	+	+	Periodogram, AR4*, AR1**
Overall MPSD	dB	+	+	Periodogram, AR4*, AR1**
Overall Percentage Distribution of EGG Power in the Bradygastria area	%	+	+	Periodogram, AR4*, AR1**
Overall Percentage Distribution of EGG Power in the Normal area	%	+	+	Periodogram, AR4*, AR1**
Overall Percentage Distribution of EGG Power in the Tachygastric area	%	+	+	Periodogram, AR4*, AR1**

AR4*, The OPSD is calculated by averaging PSDs (estimated by autoregressive modeling) of 4-minute-long segments

AR1**, The OPSD is calculated by averaging PSDs (estimated by autoregressive modeling) of 1-minute-long segments

### Computing the HRV parameters

In some case, if the signal was recorded with a relatively high sampling frequency (i.e. FS= 250 Hz), the software can be used to analyze the basic HRV parameters. These parameters are computed according the literature [1] and are summarized in Table 3. The parameters are computed for the whole examination, for 4-minute-long segments, and for the same periods as the EGG examination. An exemplary analysis of the HRV and EGG is shown in Fig. 11.

**Table 3:** The list of HRV parameters calculated by EGG DWPack Software package

Parameters	Unit	Description
RR	[*ms*]	The mean of normal RR intervals
Std RR	[*ms*]	Standard deviation of normal RR intervals
Mean HR	[1/*min*]	The mean heart rate
Std HR	[1/*min*]	Standard deviation of heart rate values
RMSSD	[*ms*]	The square root of the mean of the sum of the squares of differences between adjacent normal RR intervals
NN50	[*count*]	Number of successive RR interval pairs that differ more than 50 ms
pNN50	[%]	NN50 divided by the total number of RR intervals
VLF	[*ms*^2^]	Absolute power of VLF band (0.003–0.04 Hz)
LF	[*ms*^2^]	Absolute power of LF band (0.04–0.15 Hz)
HF	[*ms*^2^]	Absolute power of HF band (0.15–0.40 Hz)
VLF	[%])	Relative power of VLF band (0.003–0.04 Hz)
		*V* *LF*[%] = (*V* *LF*[*ms*^2^]/*totalpower*[*ms*^2^]) ∗ 100%
LF	[%])	Relative power of LF band (0.04–0.15 Hz)
		*LF*[%] = (*LF*[*ms*^2^]/*totalpower*[*ms*^2^]) ∗ 100%
HF	[%])	Relative power of HF band (0.15–0.40 Hz)
		*HF*[%] = (*HF*[*ms*^2^]/*totalpower*[*ms*^2^]) ∗ 100%
LF/HF	−	Ratio between LF and HF band powers (HRV balance)

*totalpower*[*ms*^2^] = *V* *LF*[*ms*^2^] + *LF*[*ms*^2^] + *HF*[*ms*^2^]

## Software description

The part of the EGG DWPack system for analysis the EGG examination is a MATLAB based software. The application is used for multi-channel (maximum 4 channels) EGG data conditioned with the wide range of sampling frequency and various resolutions of analog to digital (A/D) conversion. The software has been developed using MATLAB Version 7.11.0.584 (R2010b) 64-bit (win64) and also has been tested using MATLAB (R2013b).

### Input data format

**Fig. 2:**
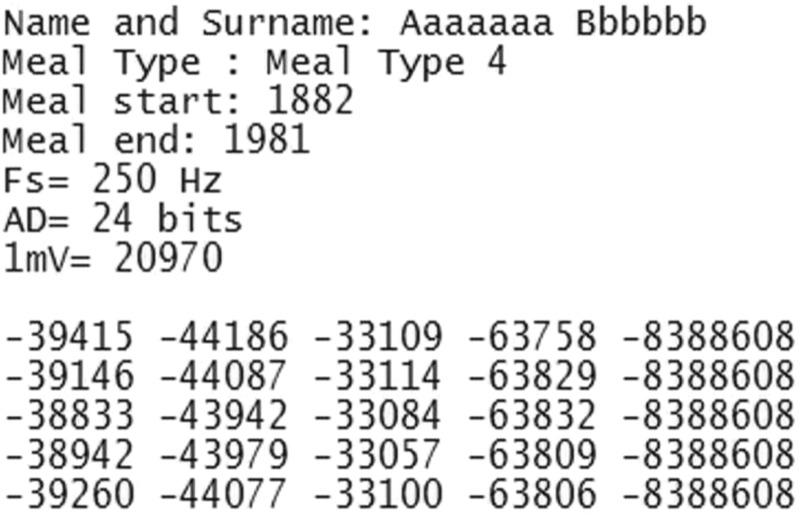
An example of the fragment of the EGG data file format

EGG DWPack requires the specific ASCII format of the EGG data file. An example fragment of the required file format is shown in Fig. 2. These files must include the 8 rows header and of course the values of the EGG data. The details of the header are summarized in Table 4. The EGG data should start in the ninth line of the file and the data of each channel should be separate by a space character. The application analyzes the 4-channel EGG data and one additional sensor channel (e.g. channel with respiration data). It is possible to analysis less than 4-channel EGG data (e.g. one channel). In this case, the application replicates the data from the first channel to the missing channels.

**Table 4:** The details of data file’s header

Number of Row	Description	Note	Unit	Example
1	patient’s data (names, code)	can be empty	-	Aaaaaa Bbbbb
2	type of meal	can be empty	a.u. (code)	Meal Type 4
3	meal start time	cannot be empty but value can be equal zero	second	start: 1800
4	meal end time	cannot be empty but value can be equal zero	second	stop: 2100
5	Sampling frequency	cannot be empty and should be >= 4*Hz*	Hz	Fs= 250 Hz
6	Resolution of analog to digital conversion	can be empty	bit	AD= 24 bits
7	Value of 1mv of the signal	cannot be empty, value of the sample for the 1mv signal	a.u.	1mV= 20970
8	empty			

### The user interface

**Fig. 3:**
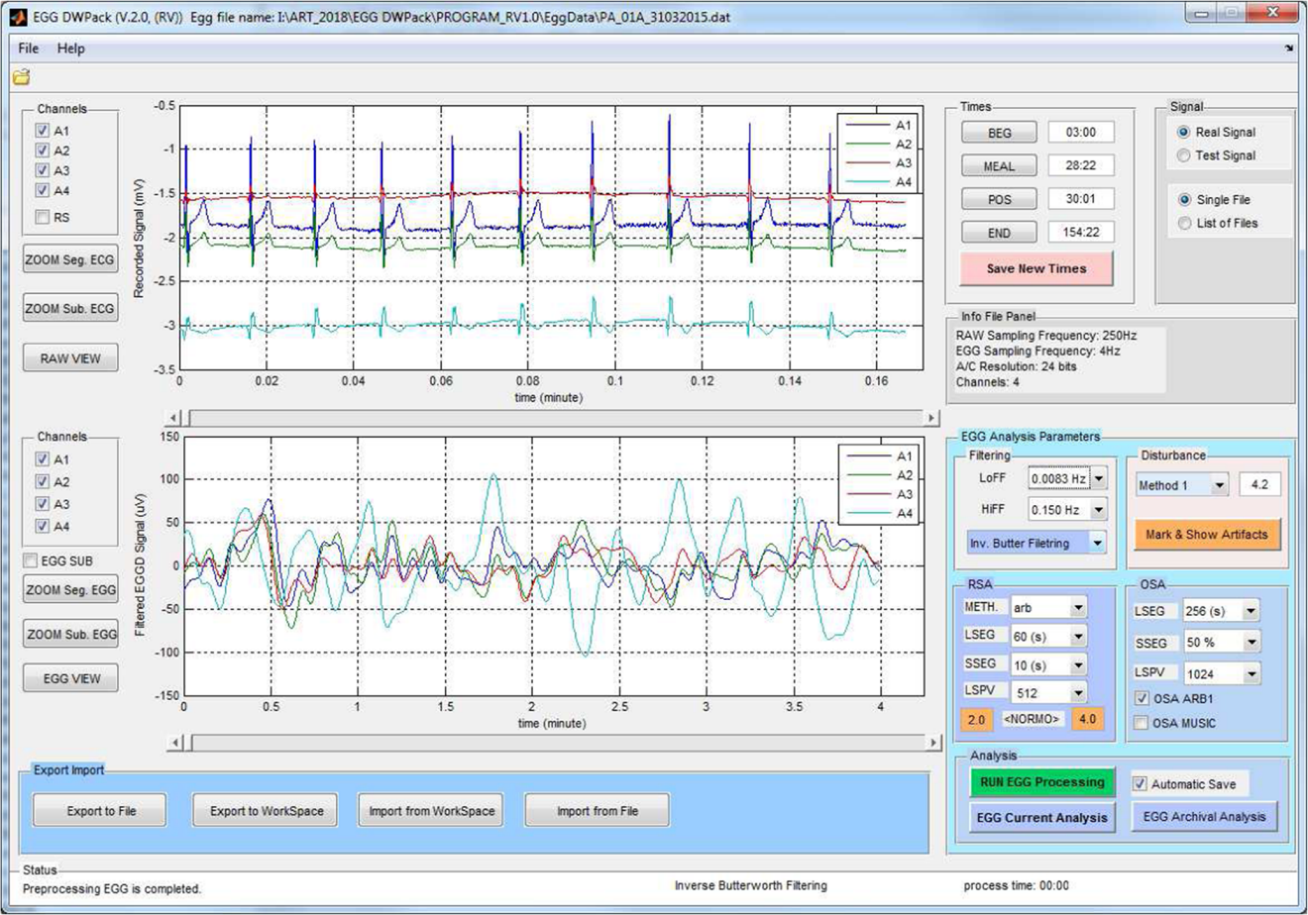
The view of user interface for browsing, preprocessing signals, and setting parameters. The upper graph shows the raw recorded signal, due to the fact that signal is sampled with *fs* = 250*Hz* the ECG (electrocardiographic) components are visible mainly. The bottom graph contains the extracted EGG components

**Fig. 4:**
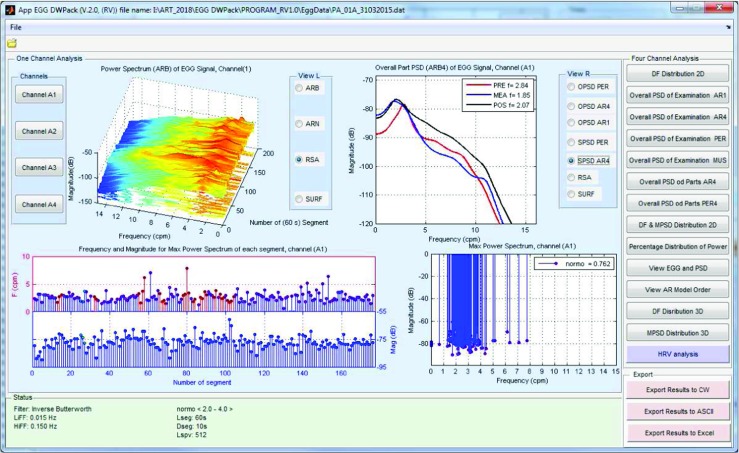
The view of user interface for browsing EGG results

The presented software package is easy to use through its user-friendly graphical interface (UFGI). The interface is multi-window and consists the parts: for browsing, preprocessing the raw (recorded) EGG signals, the analysis option settings panel and the results browser. Figure 3 shows the view of the UFGI for browsing, preprocessing signals and setting parameters. The EGG result browser is depicted in Fig. 4. The capabilities and functionalities of each of these parts are described below.

### Data browser and preprocessing signal interface

**Fig. 5:**
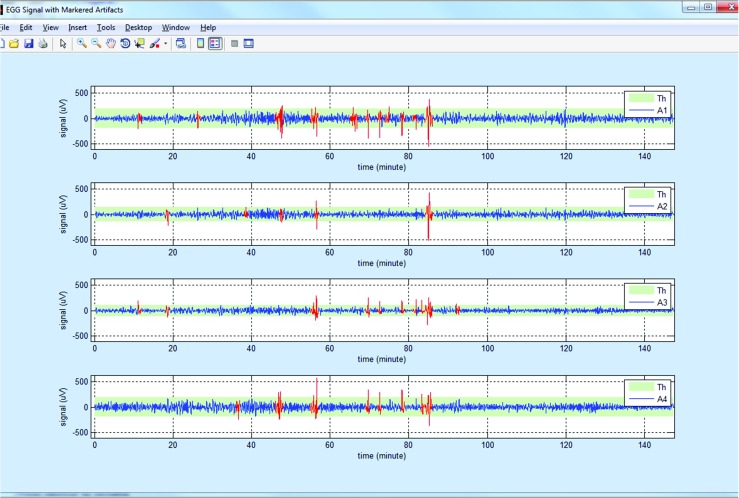
An example of recorded signals with detected artifacts (colored in red)

The data browser and preprocessing signal part (Fig. 3) consists of modules: for reading and browsing the raw (recorded) and extracted (EGG) signal, meal time correction, filtering and filtering options, artifact removal, and import and export. The EGG signal is extracted from the recorded signal using the chosen filter. When the signal is read the filtering process is applied automatically and the extracted EGG signal is shown in the lower chart (Fig. 3). If the filtering process requires changes (e.g. method or frequency range), the process can be easily repeated by choosing another filtering method or the low and the high frequencies of the filter. Because the signal can include motion artifacts caused by unavoidable body movement, the recording periods with motion artifacts should be detected and must be excluded from analysis [48]. Artifact detection can be accomplished using two different methods. The sensitivity of finding artifacts can be set by choosing an appropriate correction level (threshold for detecting artifacts). An example of a signal after artifact detection is shown in Fig. 5. The recording fragments of the EGG signal with detected artifacts are colored in red and are excluded from further analysis. If the artifact detection process requires changes (e.g. method or threshold), the process can be easily repeated by choosing another method or threshold value for detecting artifacts. In addition, the segments affected by artifacts can be manually marked (method: Manual). It can be made by editing the script file (*mArtefactsTimes*.*m*) which includes the times of beginning and the end of possible artifacts. The times should be given in minutes. The time of beginning and finishing the meal can be corrected as well. The correction can be made by typing a new value in the proper text box or by clicking signal in the top chart and next, moving the cursor, and clicking the proper button in panel *Times* (Fig. 3). That panel (*Times*) can also be used to choose the part of the signals for further processing.

**Fig. 6:**
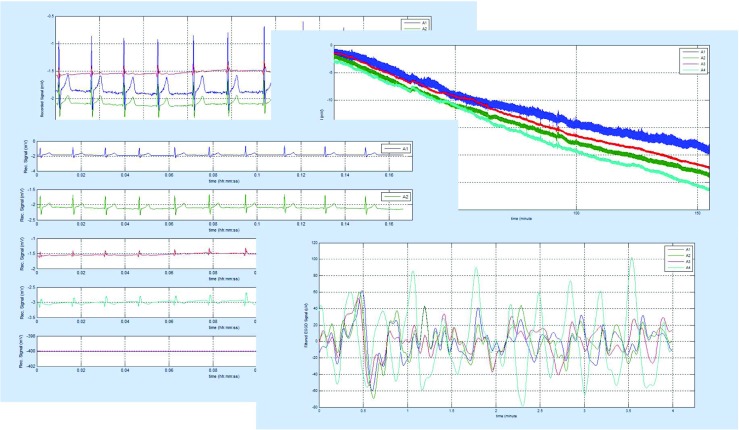
Examples of browsing the signals

The presented software can be used to browse the signal in different ways. It is possible to view raw and preprocessed signals, view all or only parts of signals, view specific channels chosen by the user or the whole examination. The recording signal and extracted EGG signal can be scrolled with the two sliders placed below the charts. Choosing the channels for browsing can be done by clicking the proper check box with the channel name. Examples of browsing views are depicted in Fig. 6.

It is also possible to export the preprocessed signal into the workspace or ASCII file and import it after additional processing. It can be made by clicking the proper buttons.

### Settings analysis, option-settings panel

This part of the presented software allows the parameters of the EGG signal analysis to be set. The user can change the parameters for the RSA and OSA analysis. For RSA, the user can set the method of spectra calculation, the length of the EGG segment (LSEG), the length of overlap (SSEG), the number of the samples of spectra (LSPV) and the range of normogastria (NORMO). For OSA, these parameters can be changed: the length of signal (LSEG) for spectral analysis, the overlap (SSEG), and the number of samples of the spectra (LSPV). All allowed settings can be made by choosing the values from the predefined settings. For OSA, it is also possible to estimate spectra by autoregressive modeling of 1-minute-long segments and the MUSIC (Multiple Signal Classification) algorithm. It can be made by selection *OSA ARB1* or *OSA MUSIC* check-box, respectively.

### EGG results browser

**Fig. 7:**
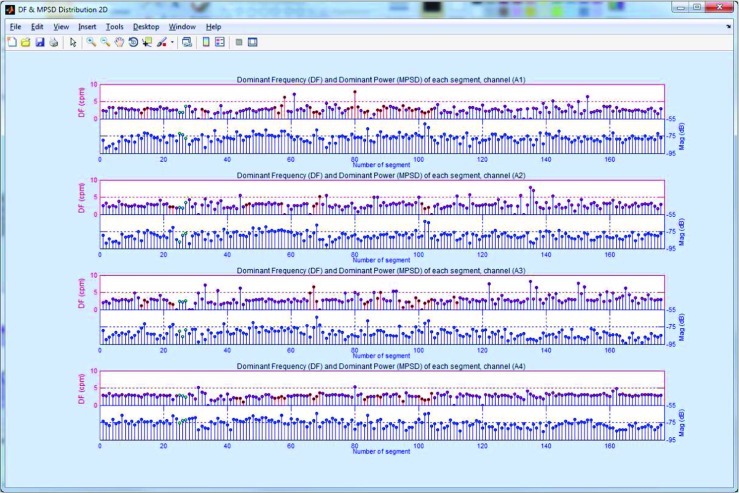
The view of dominant frequency (DF) (upper, left axis) and power (DP) (bottom, right axis) distributions for 4-channel EGG signal

**Fig. 8:**
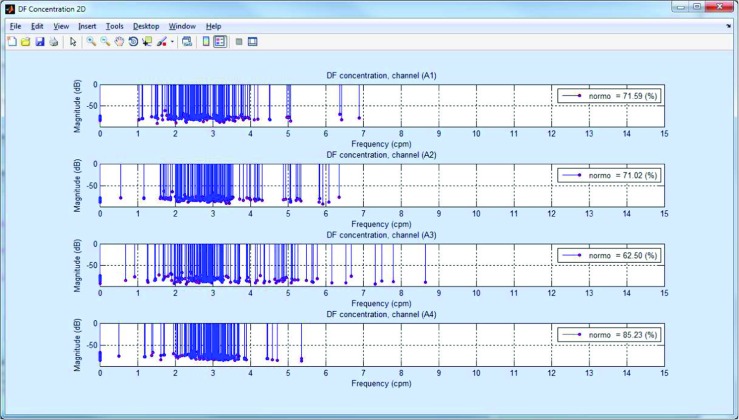
The example of the DF concentration for 4-channel EGG signal. Each blue line with magenta dot represents the DF value of the segment. The length of blue line corresponds to the value of DP (dominant power). The percentage of normal values (*normo*) are shown in upper, right corner of each subgraph

**Fig. 9:**
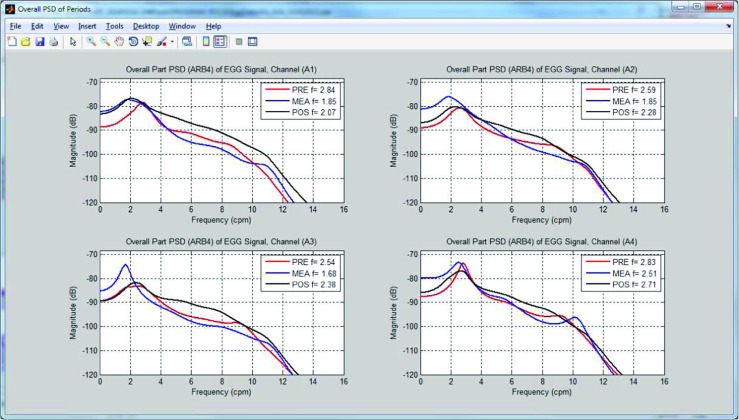
The spectra of periods and values of overall dominant frequencies for 4-channel EGG signal

**Fig. 10:**
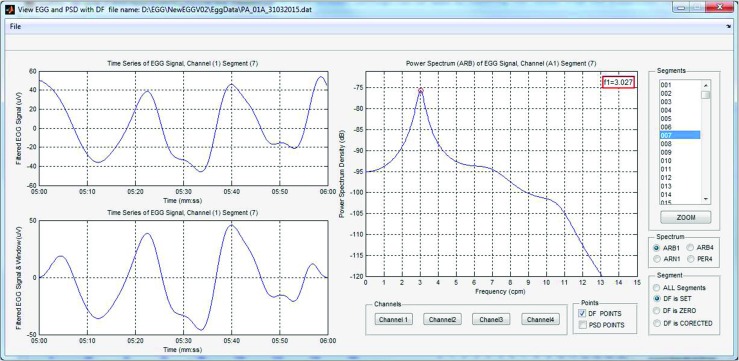
The window with the segment of EGG signal and its spectrum (interface for spectra browsing)

The EGG analysis process can be run by clicking the *Run EGG processing* button (Fig. 3). The current state of analysis is displayed in the *Status Panel* (Fig. 3). When the analysis is finished, the results are available after clicking the *EGG Current Analysis* button. It is also possible to browse the archival analysis (by clicking the *EGG Archival Analysis* button). The view of the interface for browsing EGG results is shown in Fig. 4. The results browser allows several parameters of the EGG analysis to be browsed. The main window presents the spectra of RSA and OSA analysis, the DFs, DPs (so often called MPSDs (maximum power spectrum density)), and the DF and DP distributions for the chosen EGG channel. The interface allows the observed EGG channel and the information presented in the left and right chart to be changed. The EGG analysis for all EGG channels can be made by clicking the proper button in the right panel. It is possible to view the: DF distribution, overall spectra for periods and whole examination, DFs and MPSDs, segments of EGG signal and its spectra. Figure 7 shows the results of exemplary of the DF and DP distributions. The DF concentrations are shown in Fig. 8. Figure 9 depicts the overall spectra for periods. The interface also allows a spectrum of each segment to be browsed (Fig. 10).

**Fig. 11:**
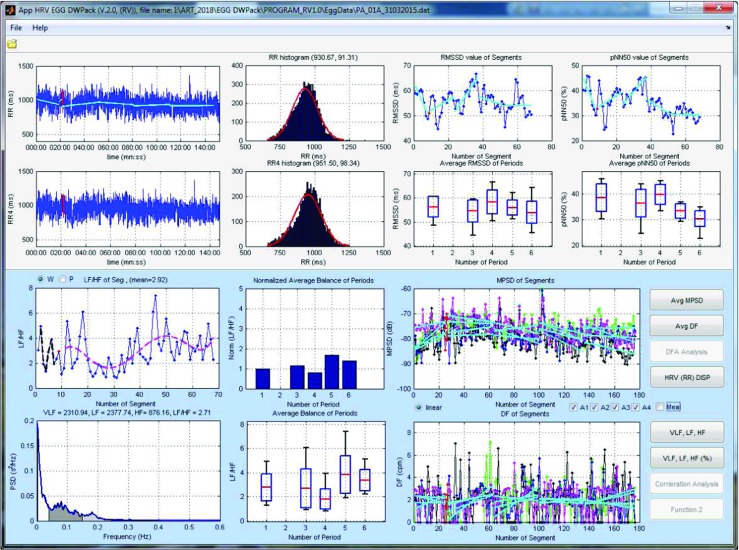
The view of the additional (optional) HRV and EGG results browser (for the high sampled EGG signal)

The interface includes the optional simultaneous analysis of EGG and HRV examinations. That analysis required an additional MAT-file, which consists of the RR intervals. That file can be obtained by using the presented MATLAB script (*mDoRRfile*.*m*). That script uses the Pan-Tompkins algorithm to detect the R peaks in QRS complexes [35] and to produce the RR interval time series. Because the RR interval time series is an irregularly sampled series, the RR values are converted into equidistantly sampled form by using cubic spline interpolation [45]. The sampling rate of the interpolation is the same for the EGG and is equal to 4 Hz. That script requires the recording signal sampled with frequency equal to 250 Hz. An additional (optional) HRV and EGG results browser is shown in Fig. 11.

### Saving the EGG results

The EGG analysis results can be automatically saved in a Matlab MAT-file. The MAT-file includes a complex structure variable named *maindata*, which includes all obtained results. The name of the saved file is created automatically and includes the name of the EGG file, and the date and time of the analysis. It is also possible to export the results to Excel and ASCII (text) files. It automatically proposes the name of the exported file, which consists of the name of analyzed EGG file, and the current date and time.

### Practical use of the EGG DWPack software, example of run

The presented software package consists of two examples of real four-channel EGG signals and four synthetic EGG signals. The real EGG data were acquired from two young healthy women. Each recording includes pre-prandial, meal and postprandial parts. The duration of recording is about two hours. The signals are sampled with frequency equal to 250 Hz and 24 bit’s resolution of A/D converter. The synthetic data are the mixture of the sinusoidal signals and noise. They are sampled with frequency equal to 200 Hz and discretized with 12 bit’s resolution. The software also includes Matlab script (*mDoArtEGGdata*.*m*) which allows simple synthetic EGG data to be produced. Using the EGG DWPack software requires MATLAB (required toolboxes: Control System, Model-Based Calibration, Signal Processing, Statistics, System Identification). The software has been tested using MATLAB Version 7.11.0.584 (R2010b) 64-bit (win64) and MATLAB (R2013b) 64-bit (win64). The software packet can be run by typing the name of the MATLAB script called *MainEGG* in *Matlab command window* and pressing *Enter*. Than the user must read the EGG data by using menu *File* or by clicking the *Open* button; after a few seconds, the EGG signal should be visible in the top chart (raw signal) and bottom chart (Fig. 3). Next, it is possible to change the parameters of preprocessing or analysis. After that, the user should press the *Run EGG Processing* button and the current state of analysis is shown in the *Status Panel*. When the analysis is finished, (it can take several seconds or minutes) in *Status Panel* the following message: *”All processes are completed, result is saved”* appears and simultaneously the background color of the *Info Panel* is changed to light violet. To view results, the user should press the *EGG Current Analysis* button. The view of the EGG results browser is shown in Fig. 4.

The user can view the chosen results by pressing the proper button or clicking the radio button. To export the results to Excel and ASCII (text) files the *Export Results to Excel* or *Export Results to ASCII* should be pressed. Additionally, the results can be displayed in the Command Window (by pressing the button *Export Results to CW* ) and could be easy written to a text file or printed.

### Availability of the software package

The software package is freely available for free of charge for Windows operating systems (MATLAB P-files and in the future like a source MAT-files) for the all possible non commercial use at: https://drive.google.com/open?id=1KnuwdVlfNUHAau9rAAoVYZBqPCiQKbq4. The *User Guide* will be shortly available too. For support please contact at the author by email: *dariusz.komorowski@polsl.pl*

## Software validation

For validating EGG DWPack, the author used synthetic and real EGG signals. The synthetic 4-channel EGG data was constructed on the assumption that the slow wave can be represented by the sine with added noise (*SNR* (signal to noise ratio) ∈{0,3,6,10} dB). The characteristic frequency can be set in the range of 0.5 to 9.0 cpm (0.008–0.15 Hz), and the amplitude is about 50–400 *μV*. The test signals were 90 minutes in length, were sampled with frequency equal to 200 Hz and discretized with 12 bits A/D resolution. The parameters of used synthetic signals and basic results of OSA analysis can be seen in Table 5.

**Table 5:** The parameters of used synthetic signals and basic results of analysis (OSA)

Settings (sine)		Calculations			
SNR	Freq.	ODF (PER)	Dif.	ODF (AR4)	Dif.
(dB)	(cpm)	(cpm)	(cpm)	(cpm)	(cpm)
10	1.50	1.50	0.00	1.51	0.01
10	3.00	3.00	0.00	2.98	−0.02
10	3.30	3.30	0.00	3.29	−0.01
10	5.40	5.40	0.00	5.39	−0.01
6	1.50	1.50	0.00	1.53	0.03
6	3.00	3.00	0.00	2.98	−0.02
6	3.30	3.30	0.00	3.29	−0.01
6	5.40	5.40	0.00	5.40	0.00
3	1.50	1.50	0.00	1.53	0.03
3	3.00	3.00	0.00	2.98	−0.02
3	3.30	3.30	0.00	3.29	−0.01
3	5.40	5.40	0.00	5.39	−0.01
0	1.50	1.50	0.00	1.57	0.07
0	3.00	3.00	0.00	2.97	−0.03
0	3.30	3.30	0.00	3.27	−0.03
0	5.40	5.40	0.00	5.39	−0.01

Except that four 4-channel synthetic signals were used to test the EGG DWPack, the validating process was performed in fourteen real human EGG data (twelve archival (Year 2009) and two new). The archival records were performed for four healthy, young subjects (all female, mean age 25.75 yr). The healthy subjects had no history of gastrointestinal diseases and were free of gastrointestinal symptoms. Body mass index (BMI) ranged from 19.8 to 21.1 *kg*/*m*^2^. The EGG data were recording with water, yogurt, and a solid meal (pancake) stimulation, respectively. Every volunteer provided a written consent to participate in that study. The research project was approved by the Bioethics Committee of the Silesian Medical University. The archival data were recorded simultaneously with the EGG Medtronic System (Polygraf ID/Polygram Net) [29]. It allows the parameters of the EGG signal analysis to be compared. The archival signals were sampled with frequency equal to 200 Hz and discretized with 12 bits A/D resolution. The new EGG data were sampled with frequency equal to 250 Hz and discretized with 24 bits A/D resolution. The mean values of the overall dominant frequency for fast, and postprandial stage were found to be 2.96±0.21 cpm (cycles per minute), and 3.05±0.33 cpm, respectively. The mean values of the normogastria index (NI) for fast, and postprandial stage were found to be 91.9±11.7 %, and 82.4±13.52 %, respectively. The mean values of slow wave coupling (SWC) for fast stage between channels A1–A2, and A1–A4 were found to be 73.59±13.43 %, and 77.54±18.98 %, respectively, whereas for postprandial stage, the mean values of SWC between channels A1–A2, and A1–A4 were found to be 66.98±18.30 %, and 48.55±13.50 %, respectively. The values established in the validation process are consistent with typical human physiological values [14, 26]. Table 6 consists of the comparison of basic EGG parameters produced by EGG DWPack and Medtronic system for pre-prandial stage. The results are similar, and the slight differences can be caused by e.g. recoding process.

**Table 6:** Comparison of basic EGG parameters produced by EGG DWPack, and medtronic system

Parameters	EGG DWPack	Medronic system	Difference
ODF (cpm)	3.0	2.89	0.11
NI (ch. A1) (%)	93.24±14.19	90.87±17.22	2.38
NI (ch. A2) (%)	84.32±19.06	79.17±19.81	5.16
NI (ch. A3) (%)	95.77± 5.85	93.09±12.51	2.68
NI (ch. A4) (%)	97.09± 4.00	94.98± 4.40	2.12
SWC (ch. A1–A2) (%)	74.03±19.19	82.51±16.04	−8.74
SWC (ch. A1–A3) (%)	77.34±23.49	86.94±18.12	−9.60
SWC (ch. A1–A4) (%)	76.67±19.81	89.17±15.26	−11.50
SWC (ch. A2–A3) (%)	68.27±23.86	80.83±19.34	−12.56
SWC (ch. A2–A4) (%)	69.79±20.36	78.05±18.51	−8.26
SWC (ch. A3–A4) (%)	80.23±15.42	89.17±11.1	−8.94

For validating HRV module, 1-hour length of the slp59 record of the MIT-BIH Polysomnographic Database [11] was used to construct the EGG and RR data. The computed results was compared against those obtained using gHRV software [43] and HRVAS software[42]. The default settings in gHRV were changed to the default settings of EGG DWPack:

- Spectrum window width: 256 s.
- Overlap: 128 s.
- Interpolation frequency: 4 Hz.
- *V* *LF*_*min*_: 0.003 Hz, *V* *LF*_*max*_: 0.04 Hz.
- *LF*_*min*_: 0.04 Hz, *LF*_*max*_: 0.15 Hz.
- *HF*_*min*_: 0.15 Hz, *HF*_*max*_: 0.40 Hz.

I compared only six parameters: pNN50, RMSSD, VLF, LF, HF and LF/HF ratio. The results of HRV analysis are very similar. They are presented in Table 7. The parameters were calculated as mean of each segment (window) (for EGG DWPAck and gHRV). The most different parameters are absolute powers of VLF, LF and HF bands. It may be caused by the method of spectrum estimation or artifacts correction method. I also compared values of VLF, LF, HF and LF/HF ratio for 4-hour length of the slp59 record to results presented in [43]. This comparison is included in Table 8. The results of validation have proved that EGG DWPack software produces reliable outcomes.

**Table 7:** Comparison of basic HRV parameters produced by EGG DWPack, gHRV, and HRVAs

Parameters	EGG DWPack	gHRV	HRVAS	Diff.1*	Diff.2**
Mean pNN50 (%)	19.38	19.54	18.80	0.16	−0.58
Mean RMSSD (ms)	42.71	42.74	43.50	0.03	0.79
Mean VLF (*ms*^2^)	3553.20	2596.76	3843.30	−956.44	290.10
Mean LF (*ms*^2^)	2232.81	1335.76	2219.80	−897.05	−13.01
Mean HF (*ms*^2^))	417.66	257.25	594.50	−160.41	168.64
Mean VLF (%)	57.63	62.63	57.70	5.00	0.07
Mean LF (%)	33.88	30.88	33.30	−4.00	−1.58
Mean HF (%))	7.48	6.96	8.90	−0.52	1.42
Mean LF/HF (a.u.)	5.53	5.57	3.73	0.04	−1.80

*Diff*.1^∗^ = (*gHRV* − *EGGDWPack*)

*Diff*.2^∗∗^ = (*HRV* *AS* − *EGGDWPack*)

RMSSD – Square root of the mean squared differences between successive RR intervals

NN50 – Number of successive RR interval pairs that differ more than 50 ms

pNN50 – NN50 divided by the total number of RR intervals

VLF(*ms*^2^)–Absolute power of VLF band (0–0.04 Hz)

LF(*ms*^2^)–Absolute power of LF band (0.04–0.15 Hz)

HF(*ms*^2^)–Absolute power of HF band (0.04–0.15 Hz)

VLF(%)–Relative power of VLF band (0.003–0.04 Hz)), *V* *LF*[%] = (*V* *LF*[*ms*^2^]/*totalpower*[*ms*^2^]) ∗ 100%

LF(%)–Relative power of LF band (0.04–0.15 Hz)), *LF*[%] = (*LF*[*ms*^2^]/*totalpower*[*ms*^2^]) ∗ 100%

HF(%)–Relative power of HF band (0.04–0.15 Hz)), *HF*[%] = (*HF*[*ms*^2^]/*totalpower*[*ms*^2^]) ∗ 100%

LF/HF–Ratio between LF and HF band powers (HRV balance.) Difference–The difference between gHRV’s value and DwPack’ value

**Table 8:** Comparison of basic frequency–domain parameters produced by EGG DWPack, gHRV, and Kubios-HRV [45]

Parameters	EGG DWPack	gHRV	Kubios-HRV
Mean VLF (%)	67.84	63.40	66.80
Mean LF (%)	21.45	26.20	22.04
Mean HF (%))	10.70	10.40	11.20
Mean LF/HF (a.u.)	2.00	2.52	1.97

VLF(%)–Relative power of VLF band

LF(%)–Relative power of LF band

HF(%)–Relative power of HF band

LF/HF–Ratio between LF and HF band powers (HRV balance)

## Discussion

In the presented paper, the author introduced the EGG DWPack software package (ver.2.0) for EGG signal analysis. The software package is easy to use through its user-friendly graphical interface. Due to the lack of standards of the EGG data, a new data format is proposed. Although only one simple file format is supported by the presented software, the software can be used for the wide range of EGG records. The conversion from any format to the proposed format should not be difficult. The software consists of various type of methods for the EGG signal analysis. The results of analysis are presented in graphic and text form. The user can set the parameters of the EGG analysis to their preferences (e.g. the range of frequency of normogastria rhythm, the length of EGG segment, the length of overlap). It is also possible to view each EGG segment and observe its spectrum. The software allows the basic parameters of the HRV signal (for EGG signal sampling with high frequency) to be analyzed as well. It allows the EGG and HRV to be simultaneously examined. The software provides the tools for preprocessing the EGG records: filtering, artifacts and noise removal, settings the time of the meal part, settings range of the analyzed signal. The software also includes the script for generating the test synthetic EGG data. The software allows calculating some parameters of the EGG examination. These parameters are calculated according to the definitions described in the literature [14, 29, 37]. It is possible to extend the range of computed EGG parameters because the majority of them are based on the spectra and values of the DF and the MPSD (DP). The presented version of the software is ready for use by the researchers and enthusiast rather than clinical users. For clinicians, the software requires further validation and creating the compiled version of proposed software, adding a module for generating and printing reports. But of course it is possible to use the software by an advanced clinical user (who know MATLAB and have basic knowledge in digital signal processing). The settings of the software options can slightly influence the obtained results (e.g. method and range of filtering, the method of computing spectra, and the length of segment, the overlap or the frequency range of normogastria). The user should be aware of these nuances.

For validating EGG DWPack, the synthetic and real EGG signals were used. The EGG software validation is a difficult task, because EGG records are not commonly and freely available (e.g. PhysioBank archives of PhysioNet (http://www.physionet.org) does not contain EGG data). The comparison to other software is also difficult due to the fact that any free available software for processing standard EGG signal is not known to author of this publication. The other limited available software is GEEMS [47]. That software is dedicated for analyzing and visualizing high-resolution (multi-electrode) recordings in spatiotemporal details. The described software will be probably the first free of charge and free available software for standard EGG data analysis. The majority parts of EGG DWPack software were validated and tested by the author over several years of dealing with the EGG signal processing [18, 19, 21, 39, 40].

### Future work

In the future author of this software is going to expand several additional functionalities e.g. add adaptive filtering [21], calculate the time shift between the slow waves in different channels [30], add the module for analysis by continuous wavelet transform based on the fast Fourier transform (CWTFFT) [19], add the module for analysis by using noise assisted multivariate empirical mode decomposition (NA-MEMD) [16], add the modules to generate and print reports, prepare the compiled version, add the possibility to save the user preferences, and any others suggested by future users of this software.
